# Disomic chromosome 3R(3B) substitution causes a complex of meiotic abnormalities in bread wheat Triticum aestivum L.

**DOI:** 10.18699/vjgb-24-42

**Published:** 2024-07

**Authors:** А.А. Zhuravleva, О.G. Silkova

**Affiliations:** Institute of Cytology and Genetics of the Siberian Branch of the Russian Academy of Sciences, Novosibirsk, Russia; Institute of Cytology and Genetics of the Siberian Branch of the Russian Academy of Sciences, Novosibirsk, Russia

**Keywords:** chromosome substitution, meiosis, FISH, immunostaining, rye Secale cereale L., common wheat Triticum aestivum L., замещение хромосом, мейоз, FISH, иммуноокрашивание, рожь Secale cereale L., мягкая пшеница Triticum aestivum L.

## Abstract

Triticum aestivum L. lines introgressed with alien chromosomes create a new genetic background that changes the gene expression of both wheat and donor chromosomes. The genes involved in meiosis regulation are localized on wheat chromosome 3B. The purpose of the present study was to investigate the effect of wheat chromosome 3B substituted with homoeologous rye chromosome 3R on meiosis regulation in disomically substituted wheat line 3R(3B). Employing immunostaining with antibodies against microtubule protein, α-tubulin, and the centromere-specific histone (CENH3), as well as FISH, we analyzed microtubule cytoskeleton dynamics and wheat and rye 3R chromosomes behavior in 3R(3B) (Triticum aestivum L. variety Saratovskaya 29 × Secale cereale L. variety Onokhoiskaya) meiosis. The results revealed a set of abnormalities in the microtubule dynamics and chromosome behavior in both first and second divisions. A feature of metaphase I in 3R(3B) was a decrease in the chiasmata number compared with variety Saratovskaya 29, 34.9 ± 0.62 and 41.92 ± 0.38, respectively. Rye homologs 3R in 13.18 % of meiocytes did not form bivalents. Chromosomes were characterized by varying degrees of compaction; 53.33 ± 14.62 cells lacked a metaphase plate. Disturbances were found in microtubule nucleation at the bivalent kinetochores and in their convergence at the spindle division poles. An important feature of meiosis was the asynchronous chromosome behavior in the second division and dyads at the telophase II in 8–13 % of meiocytes, depending on the anther studied. Considering the 3R(3B) meiotic phenotype, chromosome 3B contains the genes involved in the regulation of meiotic division, and substituting 3B3B chromosomes with rye 3R3R does not compensate for their absence.

## Abstract (ru)

У линий мягкой пшеницы с интрогрессией чужеродных хромосом создается новый генетический фон, который изменяет экспрессию генов как пшеницы, так и хромосом-доноров родственных видов. На хромосоме 3В пшеницы локализованы гены, участвующие в регуляции мейоза. Целью работы было изучить влияние замещения хромосомы пшеницы 3В гомеологичной хромосомой ржи 3R на регуляцию мейоза у дисомно замещенной линии пшеницы 3R(3B). C помощью иммуноокрашивания с антителами к белку микротрубочек, α-тубулину и центромероспецифичному гистону H3 (CENH3), а также с использованием флуоресцентной in situ гибридизации проведен анализ динамики микротрубочкового цитоскелета и поведения хромосом пшеницы и ржи 3R в мейозе линии 3R(3B) (Triticum aestivum L. сорт Саратовская 29 × Secale cereale L. сорт Онохойская). В результате работы обнаружен комплекс аномалий в динамике микротрубочек и поведении хромосом как в первом, так и во втором делениях. Особенностью метафазы I у линии 3R(3B) являлось уменьшение числа хиазм в сравнении с сортом Саратовская 29 – 34.9 ± 0.62 и 41.92 ± 0.38 соответственно. Гомологи хромосомы ржи 3R в 13.18 % мейоцитов не формировали биваленты. Хромосомы характеризовались различной степенью компактизации в 53.33 ± 14.62 клетки отсутствовала метафазная пластинка. Установлены нарушения в нуклеации микротрубочек на кинетохорах отдельных бивалентов и в их конвергенции на полюсах деления веретена. Важной особенностью мейоза было асинхронное поведение хромосом во втором делении и наличие диад на стадии телофазы II в 8–13 % мейоцитов в зависимости от изученного пыльника. Таким образом, согласно мейотическому фенотипу линии 3R(3B), на хромосоме 3В сорта Саратовская 29 находятся гены, участвующие в регуляции комплекса мейотических процессов, а замещение хромосомами ржи 3R3R хромосом 3B3B не компенсирует их отсутствия.

## Introduction

Bread wheat Triticum aestivum L. is characterized by tolerance to genomic introgressions of genetic material from wild and cultivated relatives. Alien chromosomes or their fragments may yield valuable traits, such as resistance to biotic and abiotic stresses, which is widely used in breeding programs (Mohammed et al., 2014; Yudina et al., 2014; Kroupin et al., 2019). However, introduction of alien chromosomes may also affect the regulation of basic biological processes, such as meiotic division, as early as in first-generation hybrids (Loginova et al., 2020).

Meiotic regulation in wheat has its peculiarities. While it is a hetero-hexaploid species (2n = 42, AABBDD genome), the meiotic behavior of its chromosomes matches that of a diploid organism. Chromosome pairing is controlled by Ph (Pairing homoeologous) genes. The Ph1 gene suppressing meiotic homoeologous pairing is localized on 5BL chromosome (Sears, 1977; Giorgi, 1978), and the Ph2 gene with the same albeit weaker effect, on 3DS chromosome (Mell-Sampayo, 1971). The Ph1 locus sized 2.5 МВ contains subtelomeric heterochromatin inserted within a cluster of CDK2-like genes (Griffiths et al., 2006; Al-Kaff et al., 2008; Martín et al., 2017). The gene initially referred to as “hypothetical 3” (Hyp3) (Griffiths et al., 2006; Al-Kaff et al., 2008) and later reannotated as ZIP4 (TaZIP4-B2) (UniProtKB–Q2L3T5), based on meiotic phenotype of the ph1b common wheat mutants, was incorporated into a heterochromatin segment during wheat polyploidization (Martín et al., 2017). Here, TaZIP4-B2 was responsible for progression of homologous and inhibition of homoeologous crossover, including by being involved in synaptonemal complex formation (Martín et al., 2017, 2018).

Bread wheat genome sequencing revealed the phylogenomic origin of ZIP4 (Appels et al., 2018). It was demonstrated that ZIP4 was a transduplication of a 3В chromosome locus having inparalogs on chromosomes 3A and 3D. In other words, hexaploid wheat carries four ZIP4 copies, i. e. one copy on each chromosome of group 3 (3A, 3B, 3D) and a duplicated copy on 5B chromosome. Earlier, while establishing aneuploid lines of the Chinese Spring common wheat variety, it was shown that the absence of 3B chromosome resulted in meiotic asynapsis and reduced plant fertility (Sears, 1954). The latter findings were confirmed later, and the gene was localized on the long arm of 3ВL (Bassi et al., 2013). It was shown that the loss of 3B chromosome resulted in pairing inhibition and reduced chiasmata count in meiosis. Notably, the effect of short arm (3BS) deletion was less significant than that of long arm deletion (Darrier et al., 2022). The desynapsis gene had no official designation in wheat (McIntosh et al., 2013), so, given its possible synthetic relationship to des2 on chromosome 3H in barley (Ramage, Hernandez-Soriano, 1972), the designation Tdes2 was suggested, with “des” standing for desynaptic and “T” for Triticum (Bassi et al., 2013). In addition, QTug. sau- 3B, a QTL responsible for unreduced gamete production in interspecific hybrids, was identified on 3B chromosome (Hao et al., 2014). A total of 16 meiotic genes were localized on 3B chromosome in the Chinese spring reference variety (Darrier et al., 2022). It was also shown that orthologs of wheat meiotic genes interacted with TaZIP4 of group 3 chromosomes in various meiotic processes (Alabdullah et al., 2019).

In addition to meiotic genes, there are also genes responsible for agriculturally valuable traits, such as yield, kernel weight, shape, and color, seed dormancy period, resistance to Stagonospora nodorum, Puccinia graminis f. sp. tritici, P. recondita, as well as synthesis of certain isozymes, localized on homoeologous group 3 wheat chromosomes (Munkvold et al., 2004). Qss.msub-3BL, a QTL for stem solidness, controlling sawfly resistance in bread and durum wheats, was also localized on 3BL (Cook et al., 2004). Overall, a total of 6,000 genes were localized on chromosome 3В (Paux et al., 2006). Another noteworthy discovery was the evolutionary recent (100 ka) amplification burst of LTR retrotransposons (Ling et al., 2018) capable of affecting gene structure and expression (Bariach et al., 2020). Thus, chromosome 3B substitutions or its absence become relevant in terms of hybrid genotype development. It was also shown that gene expression changes occurred in both wheat and alien chromosomes in wheat-alien addition and substitution lines (Rey et al., 2018; Dong et al., 2020).

Therefore, studying the effect of substituting wheat chromosome 3B with rye chromosome 3R on meiosis regulation in wheat-rye disomic chromosome 3R(3B) substitution line (T. aestivum L. Saratovskaya 29 variety – Secale cereale L. Onokhoiskaya variety) is a relevant research issue (Silkova et al., 2006); in the present study, we analyzed microtubule cytoskeleton dynamics and investigated meiotic cycle progression as well as the behavior of wheat chromosomes and rye chromosome 3R.

## Materials and methods

Plant material. The study employed the Saratovskaya 29 (S29) variety of Triticum aestivum L. bread wheat and wheat-rye disomic chromosome 3R(3B) substitution line (T. aestivum L. Saratovskaya 29 variety × Secale cereale L. Onokhoiskaya variety), where chromosome 3B of wheat was substituted with chromosome 3R of rye (Silkova et al., 2006) (Table 1). The plants were grown in a hydroponic greenhouse at the Institute of Cytology and Genetics, SB RAS at a 24/18 °C day/night temperature and photoperiod of LD 16:8.

Cytogenetic analysis

Acetocarmine staining. Routine study of microtubule (MT) cytoskeleton dynamics in meiosis in the S29 variety and the 3R(3B) line was performed using the technique described earlier (Loginova et al., 2020). Modified Navashin’s fluid was used as a fixative for immature spikes (Wada, Kusunoki, 1964), and meiocytes were analyzed at all stages of the first and second divisions of microsporogenesis (Table 1).

**Table 1.**
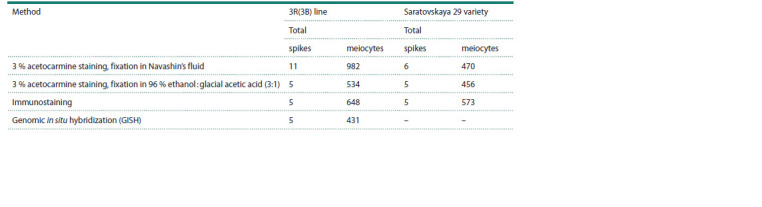
Methods used for cytogenetic analysis of the 3R(3B) line and Saratovskaya 29 plants

The specimens were studied using a Leica DM 2000 microscope (Leica Microsystems), and the images were recorded using a DFC 295 camera (Leica Microsystems).

Fluorescent in situ hybridization (FISH) and indirect immunostaining. Specimen preparation and FISH were performed using the technique described earlier (Loginova et al., 2020). Meiocytes at metaphase I and telophase II were analyzed. For the purposes of this study, we employed centromere- specific probe pAet6-09 for rice, wheat, rye, and barley chromosomes (Zhang et al., 2004), as well as genomic rye DNA. The DNA repeat sample of pAet6-09 was the courtesy of Dr. A. Lukaszewcki (University of California, Riverside, United States). Probe pAet6-09 was labeled with digoxigenin- 11-dUTP via polymerase chain reaction (PCR). The total DNA of rye was labeled with Nick-translation (Invitrogen, Carlsbad, California, United States, cat. no. 18160-010) with biotin-16-dUTP. Probes were combined in various ratios and mixed with blocking wheat DNA. To reduce fluorescence fading, Vectashield antifade solution (Vector Laboratories No. X1215) containing 1μg/ml DAPI (4′,6-diamidino- 2-phenylindol, Sigma-Aldrich, No. D9542, United States) for chromatin staining was used.

Specimen preparation and indirect immunostaining were performed using the technique described earlier (Loginova et al., 2020). The primary antibodies were anti-α-tubulin ones (Monoclonal Anti-α-Tubulin antibody produced in mouse, Sigma-Aldrich, No. T5168) (1:2,000 solution) and antibodies specific to kinetochore protein CENH3, i. e. a centromeric histone H3 variant for cereals (courtesy of Dr. A. Houben, IPK Gatersleben, Germany), 1:850 solution in 1хPBS buffer with 1 % BSA. The secondary anti-CENH3 antibodies were Rhodamine (TRITC)-conjugated AffiniPure Goat Anti-Rabbit IgG (H+L) (Jackson ImmunoResearch, No. 111-025-003) (1:100 solution); the secondary anti-α-tubulin antibodies were FITC-conjugated anti-mouse IgG (Sigma, 1:100 solution). To reduce fluorescence fading, we applied Vectashield antifade solution (Vector Laboratories No. X1215) containing 1 μg/ ml DAPI (4′,6-diamidino-2-phenylindol, Sigma-Aldrich, No. D9542, United States) for chromatin staining.

The specimens were studied using an Axio Imager M1 microscope (Carl Zeiss AG, Germany) with ProgRes MF camera (Meta Systems, Jenoptic, Germany), Isis imaging software (Meta Systems, Jenoptic, Germany) as well as a LSM 780 NLO laser scanning microscope (Zeiss) with an AxioCam MRm camera (Zeiss) and ZEN imaging software (Zeiss). The images obtained were processed in Adobe Photoshop CS2

## Results

Chromatin and microtubule cytoskeleton dynamics at prophase of the first meiotic division in the S29 variety and the 3R(3B) line

Comparative analysis of prophase progression in the S29 variety and the 3R(3B) line did not show any differences before zygotene (Supplementary Materials 1 and 2)1. Meiocytes in the S29 variety and the 3R(3B) line changed their shape from rectangular (Supplementary Materials 1a and 2a, d ) and triangular (Supplementary Materials 1b and 2c) to rounded (Supplementary Materials 1d and 2b) starting with early leptotene.

**Fig. 1.**
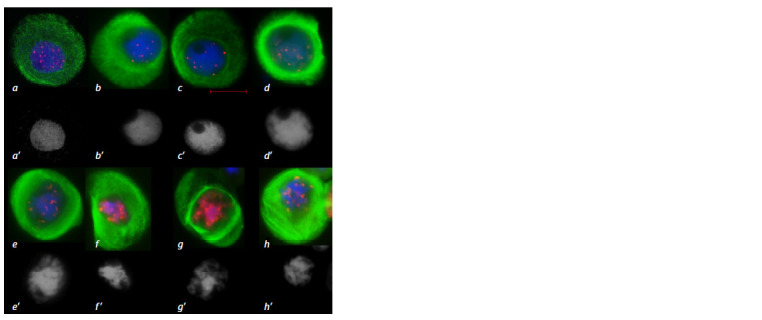
MT cytoskeleton reorganization at prophase of the first meiotic division in the 3R(3B) line. a – leptotene-zygotene, prophase reticular cytoskeleton; b – zygotene, MTs move toward the nucleus to form a perinuclear ring; c – pachytene, dense MT ring; d – diplotene, MTs form a dense ring around the nucleus; e–h – diakinesis, consecutive stages of ring disintegration and MT reorientation; l – pro-spindle formation. Immunostaining. DNA is shown in blue, MTs in green, centromeric kinetochores in red; scale bar length is 5 μm. a’–h’ – DAPI staining.

Three to four nucleoli were present in early leptotene (Supplementary Materials 1a, b and 2b) to later fuse into one (Supplementary Materials 1c, d and 2e). In leptotene-zygotene, thin chromatin threads formed a dense ball containing a single nucleolus shifted toward the nuclear envelope (Supplementary Materials 1d and 2e; Fig. 1a′, b′). Meiocyte maturation was accompanied by chromatin condensation. In zygotene, chromatin fiber thickening was observed (Supplementary Materials 1d and 2e–g).


Supplementary Materials are available in the online version of the paper: https://vavilov.elpub.ru/jour/manager/files/Suppl_Zhuravleva_Engl_28_4.pdf


In zygotene and pachytene, the S29 variety and the 3R(3B) line showed different chromatin distribution across the nucleus. Compared to the S29 variety, the 3R(3B) line was cha-racterized by radial and irregular chromatin looping (Supplementary Material 2i, j). In pachytene, asymmetric chromosome grouping on one side of the nucleus was observed in both wheat and wheat-rye substitution line (Supplementary Materials 1e and 2h). In diplotene, chromatin threads were shortened even more, while still in contact with the nuclear envelope (Supplementary Materials 1f, g; and 2l). In diakinesis, bivalent formation was com-pleted with the nucleolus and the nuclear envelope still present (Supplementary Materials 1h and 2m, n). Compared to the S29 variety, chromosomes were densely packed in the 3R(3B) line in diakinesis (Supplementary Materials 1h and 2n).

Immunostaining analysis of meiocytes at prophase before pachytene did not show any differences between the MT dynamics observed in the 3R(3B) line and the earlier results for S29 (Loginova et al., 2020). Reticular cytoskeleton formation was observed at interphase and early prophase (Fig. 1a), then MTs were reorganized into radial bundles, reoriented, and moved toward the nucleus in zygotene- pachytene, while the nucleus itself migrated toward the envelope to form a “half-moon” MT structure (Fig. 1b).

**Fig. 2.**
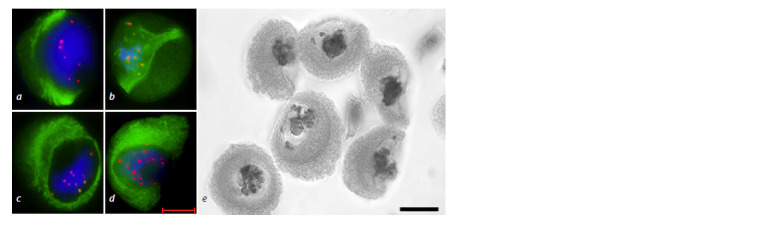
Migration of the nucleus to the cell periphery and formation of arc-like MT structures at meiotic prophase in the 3R3B line. a, d – arc formed by MTs; b – MTs at the top of the arc form a spindle pole-like structure; c – incomplete migration of the nucleus to the periphery; e – a group of cells partially demonstrating migration of the nucleus toward the meiocyte envelope. Immunostaining (a–d ). DNA is shown in blue, MTs in green, centromeric kinetochores in red; scale bar length is 5 μm. Navashin’s fluid fixation (e), acetocarmine staining; scale bar length is 10 μm.

In pachytene, a dense perinuclear ring was formed around the nucleus (Fig. 1c) at the center of the cell. The nucleus migrated toward the cell envelope, and cytoskeleton formed an arc-like structure in 10–90 % of the cells depending on the anther studied (Fig. 2a, c, d ). MT nucleation density in the latter varied. In some meiocytes, MTs at the top of the arc formed a spindle pole-like structure (Fig. 2b).

In diplotene-diakinesis, ring disintegration occurred, and MTs were separated into beams and straightened out (Fig. 1e– g), 3- and 4-pole structures were formed in diakinesis (Fig. 1g, h) in both S29 variety wheat and rye (Loginova et al., 2020).

**Fig. 3.**
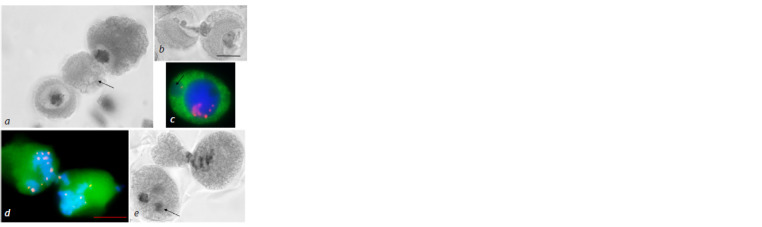
Cytomixis in the wheat-rye 3R(3B) substitution line. a – migration of the nucleus at prophase with cytoplast formation (arrow); b – chromatin transfer from one meiocyte to another; c – micronucleus (arrow); d – chromosome transfer from one meiocyte to another at late prophase; e – cytomixis at metaphase I. Navashin’s fluid fixation, acetocarmine staining (a, b, e); scale bar length is 10 μm. Immunostaining (c, d ). DNA is shown in blue, MTs in green, centromeric kinetochores in red; scale bar length is 5 μm.

Migration of the nucleus toward the membrane in 5 % of the cells in the 3R(3B) line ended with chromatin transfer from one meiocyte to another as a result of cytomixis (Fig. 3; Table 2). Chromatin transfer occurred at prophase (Fig. 3a, b, d ) and metaphase I (Fig. 3e). The cells chromatin was transferred from had reduced chromatin content or formed cytoplasts (Fig. 3a). The transferred chromatin formed a separate micronucleus (Fig. 3c) or was fused into the nucleus of the recipient cell (Fig. 3e).

**Table 2.**
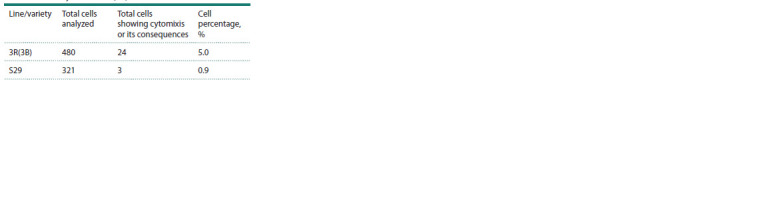
Cytomixis frequency in the S29 variety and the 3R(3B) line

Chromosome behavior and MT cytoskeleton dynamics in the first meiotic division in the S29 variety and the 3R(3B) line

**Fig. 4.**
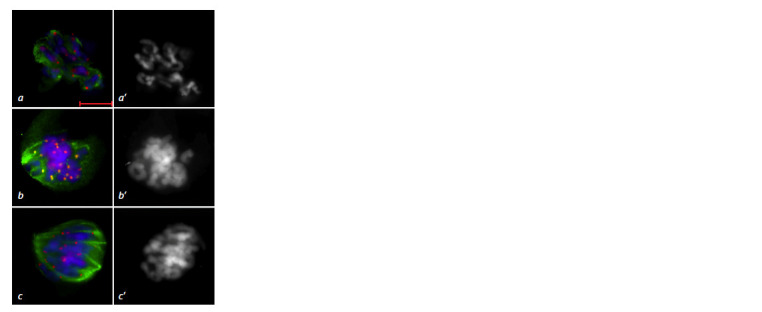
Meiotic prometaphase I in the 3R(3B) line. a – interaction between MTs and chromosome kinetochores; b, c – formation of central and kinetochore fibrils for the spindle apparatus Immunostaining. DNA is shown in blue, MTs in green, centromeric kinetochores in red; scale bar length is 5 μm. DAPI staining (a’–c’).

After nuclear envelope disintegration, prophase spindle was disassembled, and at prometaphase MTs interacted with chromosome kinetochores and with each other to form central and kinetochore fibrils for the spindle apparatus in both the S29 variety and the 3R(3B) line (Fig. 4).

**Fig. 5.**
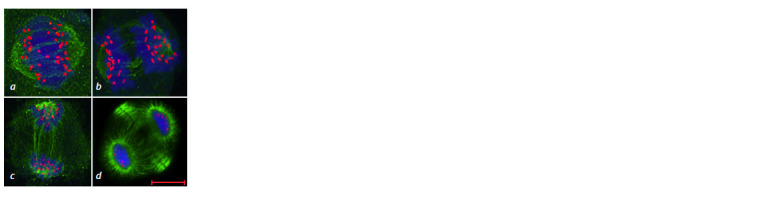
MT cytoskeleton reorganization in the first meiotic division in the 3R(3B) line. a – metaphase, spindle apparatus is formed; b – anaphase, chromosomes are pulled to the poles with shortening of kinetochore fibrils of the spindle apparatus; c – late anaphase, only central fibrils of the spindle apparatus are present, radial cytoskeleton formation is initiated; d – telophase, phragmoplast formation Immunostaining. DNA is shown in blue, MTs in green, centromeric kinetochores in red; scale bar length is 5 μm.

The ends of the microtubules converged at the poles to form the spindle apparatus and chromosomes were aligned along the equator of the cell (Fig. 5a).

A distinctive feature of metaphase I in the 3R(3B) line is the absence of the metaphase plate at the equator of the spindle apparatus and a different degree of chromosome compaction (Supplementary Material 3; Fig. 13). Meiocytes lacking a metaphase plate add up to 20 to 100 % depending on the anther analyzed, the average being 53.33 ± 14.62 % (Supplementary Material 3a–e).

**Fig. 6.**
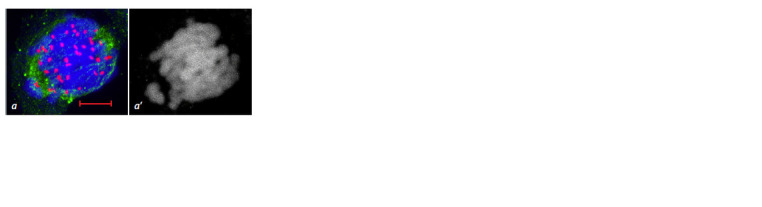
Absence of the metaphase plate at metaphase I in the 3R(3B) line. Immunostaining. DNA is shown in blue, MTs in green, centromeric kinetochores in red; scale bar length is 5 μm. DAPI staining (а’).

**Fig. 7.**
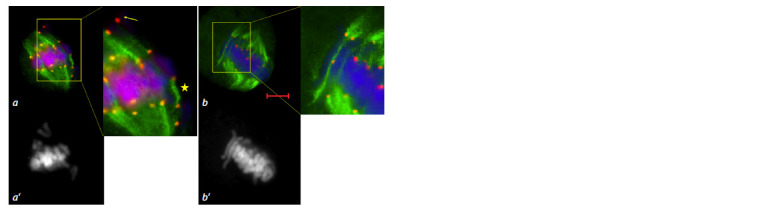
Disruption of MT nucleation during spindle apparatus formation at metaphase I. a – absence of MT nucleation at the kinetochore of a rod bivalent (arrow), MT beams connect kinetochores of rod and ring bivalents (star); b – bivalents lacking MT convergence at the poles. Immunostaining. DNA is shown in blue, MTs in green, centromeric kinetochores in red; scale bar length is 5 μm. DAPI staining (a’, b’).

**Fig. 8.**
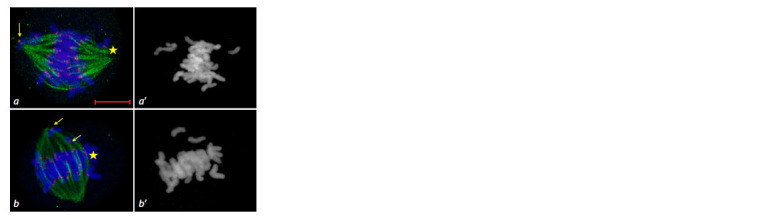
Univalent distribution at metaphase I in the 3R(3B) line. a – MT attachment of univalents to one pole (star), absence of α-tubulin signal at the bivalent kinetochore (arrow); b – absence of α-tubulin signal at the univalent kinetochore (arrows), bipolar orientation of separated sister kinetochores (star). Immunostaining. DNA is shown in blue, MTs in green, centromeric kinetochores in red; scale bar length is 5 μm. DAPI staining (a’, b’).

Immunostaining analysis of chromosome behavior in the 3R(3B) line showed chaotic distribution of bivalents along the equatorial plane in cells lacking a metaphase plate (Fig. 6a) compared to the normal case (Fig. 5a), due to the absence of MT nucleation at kinetochores of individual bivalents (Fig. 7a, 8a) or anomalous connection of kinetochores of open and closed bivalents by MT beams (Fig. 7a). Meiocytes, where normal spindle apparatus could not be formed due to the absence of MT convergence at the pole (2 % meiocytes), were observed (Fig. 7b).

Metaphase I in the 3R(3B) line is characterized by reduced chiasmata count compared to the S29 variety (Table 3; Supplementary Material 3). According to observations, the number of rod bivalents per cell was 3.0 ± 0.35, the number of ring bivalents per cell was 15.95 ± 0.61, and the number of univalents was 3.79 ± 1.0 (Table 3). No univalents were observed in the S29 variety, the number of ring bivalents was 20.92 ± 0.04, and the number of rod bivalents was 0.08 ± 0.04 (Table 3). Multivalents were observed in 1.2 % of meiocytes in the 3R(3B) line.

**Table 3.**
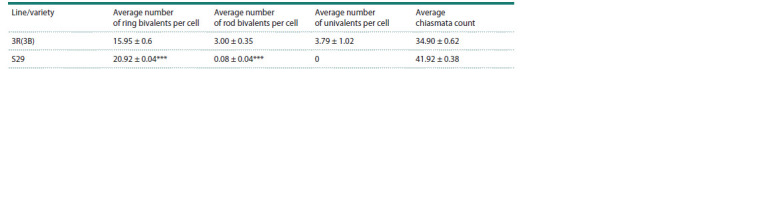
Formation of bivalents and univalents in the 3R(3B) line and the S29 wheat variety *** Significant differences at p ≤ 0.001.

Sister kinetochores of univalent chromosomes at metaphase I were either separated or remained fused. In the first scenario, chromosomes were aligned along the equator (Fig. 8b), and in the second one, they were pulled randomly towards the poles before anaphase I (Fig. 8a). The absence of MT nucleation at the single kinetochore of a univalent (Fig. 8b) and a bivalent (Fig. 8a) could also cause metaphase plate formation abnormalities

At early anaphase I, kinetochore fibrils of the meiotic spindle were shortened, and chromosomes were pulled to the poles in both the S29 variety and the 3R(3B) line (Supplementary Materials 4b, c and 5b, c). Chromosome distribution across the spindle apparatus did not depend on the degree of compaction (Supplementary Material 5b). Chromosome separation was followed by formation of a phragmoplast-cell plate structure (Supplementary Materials 4d and 5d ) dividing a meiocyte into two daughter cells. The first division ends with the formation of a dyad with a radial cytoskeleton (Supplementary Materials 4e and 5e).

**Fig. 9.**
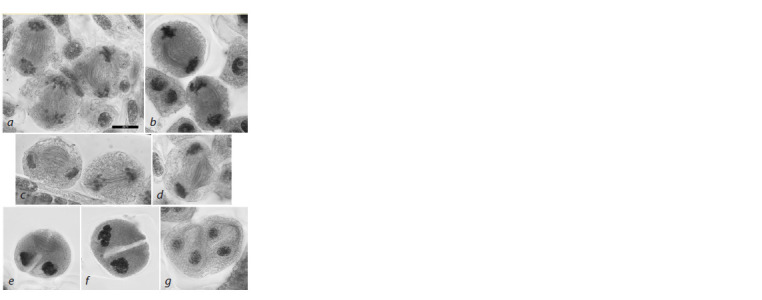
C-shaped spindle formation in the 3R(3B) line. a–c – anaphase I; d – telophase I, autonomous spindle orientation; e, f – telophase I, cell wall in the form of a notch; e – autonomous spindle orientation; g – telophase II Navashin’s fluid fixation, acetocarmine staining; scale bar length is 10 μm.

C-shaped spindles not affecting chromosome separation were observed at metaphase I and anaphase I (0 to 30 % of meiocytes, depending on the anther) and at telophase II in the 3R(3B) line (Fig. 9a–d, g).

The spindle apparatus maintained its shape after chromosome separation and was located near two telophase chromosome groups (Fig. 9c–e). Phragmoplast formation was disrupted, and cell wall emerged in the form of a notch not ensuring full separation of a meiocyte at telophase I (Fig. 9e, f ).

Second meiotic division in the S29 variety and the 3R(3B) line

Analysis of the second division showed the presence of anthers with abnormalities in addition to normal meiotic progression in the 3R(3B) line similarly to the first division as opposed to the S29 variety. Normally, the radial cytoskeleton (Supplementary Material 6a) was transformed into MT beams at the second division prophase. The latter then formed the metaphase structure (Supplementary Materials 6b, c and 7b, c) and distributed sister chromatids between the poles (Supplementary Materials 6d and 7d ).

**Fig. 10.**
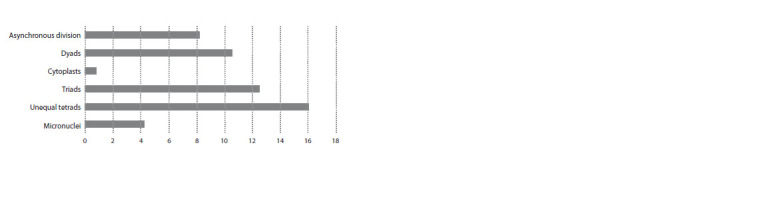
Percentage of cells with various anomalies in the second meiotic division in the 3R(3B) line.

Similarly to the first division, phragmoplast-cell plate system is formed, central fibrils of the spindle apparatus are preserved, and the division ends with tetrad formation (Supplementary Materials 6e, f and 7e, f ). Micronuclei were detected in 4.2 % of tetrads (Fig. 10).

**Fig. 11.**
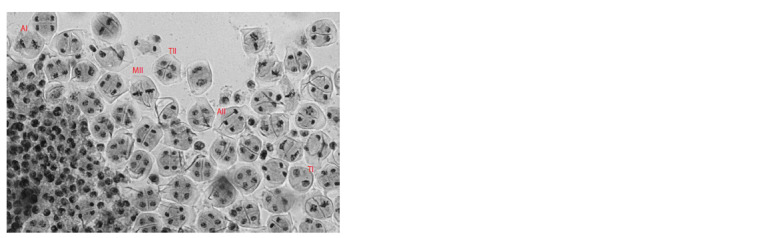
Asynchronous chromosome behavior in the 3R(3B) line. Cells at different meiotic division stages within the same anther. AI – anaphase I, TI – telophase I, MII – metaphase II, AII – anaphase II, and TII – telophase II. Navashin’s fluid fixation, acetocarmine staining.

Asynchronous chromosome behavior was observed in the second division in the 3R(3B) line (Fig. 10, 11). Certain anthers from the same spike can simultaneously include meiocytes at different division stages: anaphase I, telophase I, metaphase II, anaphase II, and tetrads (Fig. 11). Dyads can be observed among the tetrads at telophase II (8 to 13 % of meiocytes per anther studied) (Fig. 10).

**Fig. 12.**
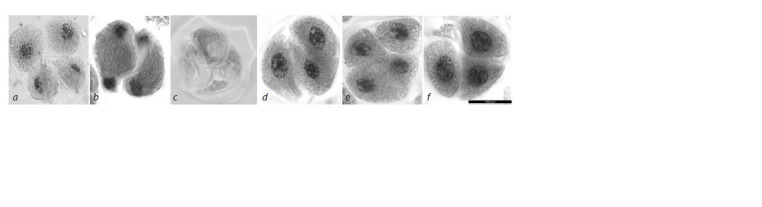
Tetrad stage anomalies in the second meiotic division in the 3R(3B) line. a – tetrad with unequally sized nuclei; b – anomalous spindle in the second division, chromatin imbalance; c – tetrad without a nucleus; d, f – triads; e – absence of cell wall in one out of two cells. Navashin’s fluid fixation, acetocarmine staining; scale bar length is 10 μm.

At telophase II, tetrads with unequally sized nuclei were observed in 10–20 % of meiocytes (Fig. 10, 12a), cytoplasts without nuclei, in 2.4 % of cells (Fig. 10, 12c), and triads, in 12.5 % cells (Fig. 10, 12d–f ).

Rye chromosome 3R3R behavior in the first and second meiotic divisions

**Fig. 13.**
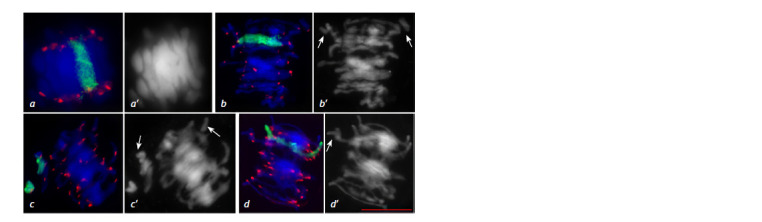
Chromosome behavior at meiotic metaphase I in the 3R(3B) line. a – ring bivalent formation by chromosomes 3R3R, normal chromosome alignment along the equator; b–d – disturbance of chromosome compaction; b – ring bivalent formation by chromosomes 3R3R, wheat chromosome univalents (arrows); c – rye chromosome univalents, wheat chromosome univalents (arrows); d – open bivalent formation by chromosomes 3R3R, wheat chromosome univalent (arrow). GISH: DNA is shown in blue, rye chromosomes in green, centromeric region in red. Scale bar length is 5 μm.

Rye chromosome 3R3R behavior was studied using FISH. At metaphase I, chromosomes 3R3R formed bivalents in 86.82 % of meiocytes, among which 21.36 % were rod bivalents and 13.18 % were univalents (Fig. 13). Chromosome 3R was absent in 5.58 % of the cells.

**Fig. 14.**
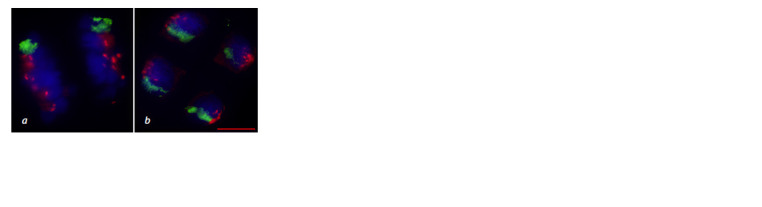
Meiotic distribution of rye chromosomes in the 3R(3B) line at telophase I (a) and telophase II (b). GISH: DNA is shown in blue, rye chromosomes in green, centromeric region in red. Scale bar length is 5 μm.

At telophase I, separation of homologous chromosomes 3R3R was not disrupted in 98 % of meiocytes (Fig. 14a). At telophase II, the analysis showed the presence of chromosome 3R in all microspores of the tetrad (Fig. 14b), which is indicative of normal distribution pattern for both bivalents and univalents

## Discussion

Chromosome 3B is required for chiasmata formation between homologs, and its absence is not compensated by rye chromosome 3R

Chiasmata formation between homologs with simultaneous suppression of chiasmata formation between homoeologous chromosomes in bread wheat is controlled by the TaZIP4-B2 gene identified within the Ph1 locus localized on the long arm of chromosome 5B (Griffiths et al., 2006; Al-Kaff et al., 2008; Martín et al., 2017). However, cytogenetic studies of microsporogenesis in mutants of tetraploid and hexaploid wheats, as well as their aneuploid and deletion lines showed that genes regulating bivalent formation were also localized on wheat chromosome 3B independently from 5B (Sears, 1954; Lee et al., 1970; Lelley, 1976; Miller et al., 1983; Darrier et al., 2022; Draeger et al., 2023). For instance, nullisomic chromosome 3B in hexaploid wheat in presence of two 5В chromosomes causes reduced chiasmata count at metaphase I (asynapsis) (Sears, 1954; Lee et al., 1970; Kato, Yamagata, 1982; Darrier et al., 2022), while long arm deletions of varying sizes of chromosome 3B reduce the total chiasmata count by 35 % (Darrier et al., 2022).

Our study has shown that the distinctive feature of metaphase I in the 3R(3B) line is the reduced chiasmata count compared to the S29 variety, 34.9 ± 0.62 and 41.92 ± 0.38, respectively. Homologs of rye chromosome 3R3R also form bivalents only in 86.82 % of meiocytes, among which 21.36 % are rod bivalents. The earlier analysis of chromosome composition in the 3R(3B) line using cytogenetic and molecular methods showed the presence of two 5B chromosomes in the karyotype (Silkova et al., 2006). Thus, our results confirm the earlier findings with regard to the presence of genes on chromosome 3В regulating chiasmata formation independently from genes on chromosome 5В.

It has been recently confirmed that the ZIP4 gene copies in the Ph1 locus on chromosomes 5В (TaZIP4-B2), 3A (TaZIP4-А1), 3B (TaZIP4-B1), and 3D (TaZIP4-D1) do not compensate for the absence of each other (Rey et al., 2017; Draeger et al., 2023). The absence of TaZIP4-B2 expression in ethyl methanesulfonate-induced TILLING Ph1 mutants does not cause an equivalent increase in the expression of ZIP4 homologs on homoeologous group 3 chromosomes (Rey et al., 2017). Cytogenetic analysis of chiasmata formation in TILLING mutants focusing on three copies of ZIP4 genes in tetraploid wheat has shown that Ttzip4-A1 produced a phenotype that is almost identical to wild wheat (Draeger et al., 2023). Significant reduction in the chiasmata count by 10 % occurs in the Ttzip4-B1 and Ttzip4-B2 single mutants, as well as in the Ttzip4-A1B2 and Ttzip4-B1B2 double mutants, but the differences between them are insignificant with only an average of 1–2 extra univalents per cell (Draeger et al., 2023). Crossovers in the Ttzip4-A1B1 double mutants (with a single TtZIP4-B2 copy) are reduced by 76–78 %, and the plants frequently become sterile (Draeger et al., 2023). The TaZIP4 copies on group 3 chromosomes are also predominantly required for homologous crossovers in hexaploid wheat (Martín et al., 2021).

A set of genes is identified on chromosome 3В, of which at least eight (CAP-E1/E2, DUO1, MLH1, MPK4, MUS81, RTEL1, SYN4, ZIP4) were confirmed to be involved in recombination process (Darrier et al., 2022). Three copies of genes CAP-Е1/E2, MLH1, and MPK4-3 were characterized by the highest expression levels, while ZIP4 expression level was significantly lower or equal to that of 3A, 3B, and 3D homoeologs. As a result, MPK4, CAP-Е1/E2, and MLH1 were picked as candidate genes responsible for chiasmata formation control (Darrier et al., 2022).

Another distinctive feature of metaphase I in the 3R(3B) line was the presence of meiocytes with decompacted chromosomes. The AtCAP-E1+/– and AtCAP-E2–/– heterozygous double mutants of Arabidopsis turned out to be the closest ones in terms of meiotic phenotype, where the CAP-Е1/E2 gene acted like a functional ortholog of the SMC2 (Structural Maintenance of Chromosomes 2) gene, a subunit of the condensin complex involved in chromosome compaction (Sutani et al., 1999). The analysis of mutants showed the expression of these genes during meiosis, and heterozygous double mutants demonstrated reduced chromosome condensation at metaphase I and anaphase I (Siddiqui et al., 2003). Some authors (Darrier et al., 2022) consider anomalous condensin activity an additional factor contributing to crossover disruption.

The presence of meiotic genes on chromosome 3B was further proved by QTL mapping of QTdes2.ndsu-3B responsible for desynapsis in durum wheat plants with chromosome 3B long arm deletion caused by radiation exposure (Bassi et al., 2013). However, the nucleotide sequence for this deletion has not been sequenced to date and cannot be compared to the sequences of the known genes.

Our study has also demonstrated that rye chromosome 3R does not compensate for the ability of chromosome 3B to ensure normal formation of crossovers between homologs. Asynapsis between homologs as a result of chromosome 3В substitution with wheat or rye homoeologs was demonstrated earlier (Lee et al., 1970; Bassi et al., 2013). Up to 14 univalents were formed in a 3D(3B) substitution line of durum wheat, the Langdon variety, at metaphase I (Bassi et al., 2013). Substitution of wheat chromosome 3В with rye chromosome 3R in the Kharkovskaya-Dakold bread wheat line caused asynapsis between homologs in 30 % of meiocytes (Lee et al., 1970). However, the addition of a pair of rye chromosomes 3R into the karyotype of F1 wheat-rye hybrids increased the number of bivalents at metaphase I (Lelley, 1976; Miller et al., 1983), while the lowest reduction of chiasmata count of 1.1 % was produced by chromosome 3R in the Chinese Spring-Imperial addition line (Orellana et al., 1984).

Chromosome 3R(3B) substitution causes various meiotic division abnormalities

Meiosis in the 3R(3B) line was characterized by a number of abnormalities in MT dynamics and chromosome behavior in the first and second divisions. These results can be explained by the earlier data on the co-expression of Ttzip4-B1 and meiotic genes orthologs (Alabdullah et al., 2019). During the construction of co-expression network for the orthologs of known meiotic wheat genes associated with TaZIP4, three TaZIP4 homoeologs on group 3 chromosomes 3A, 3B, and 3D (TraesCS3A02G401700, TraesCS3B02G434600 and TraesCS3D02G396500) were clustered in the largest meiosisrelated module and significantly linked to many orthologs of meiotic genes with various functions as follows: association of sister kinetochores in the first meiotic division, chromosome segregation, formation of class I and II crossovers, protection of the cohesin complex in the centromeric region, control of the meiotic cell cycle, sister chromatid cohesion, double-strand break DNA repair, synaptonemal complex, anti-crossover activity, and double-strand break formation in DNA (Alabdullah et al., 2019). However, the TaZIP4 copy responsible for the Ph1 phenotype (TraesCS5B02G255100) was not clustered in the same module (Alabdullah et al., 2019), which also confirms its alternative expression profile (Martín et al., 2018).

Our study has discovered anomalies in MT cytoskeleton dynamics in the 3R(3B) line. At metaphase I, we observed the disruptions in MT nucleation at kinetochores of certain bivalents or МТ convergence at the pole, which could cause the absence of equator plate in 53.33 ± 14.62 % of meiocytes. We also observed the formation of an arc-like structure by the cytoskeleton, when the nucleus migrated toward the nuclear envelope at pachytene. A possible cause for that could be the absence of the MPK4 (mitogen-activated protein kinase) gene identified on chromosome 3В (Darrier et al., 2022) and involved in MT cytoskeleton dynamics (Beck et al., 2010; Zheng et al., 2011).

Asynchronous chromosome behavior in the second division and the presence of dyads at telophase II was a significant meiosis feature in the 3R(3B) line. This meiotic phenotype matched ТАМ mutants (tam 1, tam2), where tam1 demonstrated asynchronous meiotic division, and tam2, the absence of the second division and subsequent meiotic restitution. QTug.sau-3B, a QTL responsible for unreduced gamete production in interspecific hybrids, was identified on chromosome 3B (Hao et al., 2014) and turned out to be syntenic for the ТАМ locus in rice and Brachypodium, while in Arabidopsis thaliana, ТАМ codes for CYCA1;2 cyclin.

The absence of wheat chromosome 3B is not the only possible cause of meiotic division disturbances in the 3R(3B) line. At present, changes in gene expression levels have been detected both in wheat-alien chromosome substitution and addition lines (Rey et al., 2018; Dong et al., 2020). Disturbances in chromosome behavior in the bread wheat lines introgressed with alien chromosomes are made possible due to the formation of a new genetic background where gene expression levels change in both wheat recipients and alien donors (Rey et al., 2018; Dong et al., 2020). For instance, changes in gene expression levels were detected in all wheat chromosomes in the TA3575 line where chromosome 3В was substituted with 3Sl#2 of Ae. longissima (Dong et al., 2020). Transcriptome analysis showed changes in gene expression in 577 out of 1,839 genes mapped on chromosome 3В of the Chinese Spring variety (31.43 %). Most of these genes (461, 79.90 %) were not transcribed, and 100 genes (17.33 %) demonstrated reduced expression, whereas only 16 (2.77 %) genes showed increased expression. It shows that at least 34.57 % (461 out of 1,839) of the genes on the absent chromosome 3B were not genetically compensated for by introgression of chromosome 3Sl#2 of Ae. longissimi (Dong et al., 2020).

## Conclusion

Introgression of genetic material from relatives in the form of chromosomes or their fragments into bread wheat genome is widely used in wheat breeding to transfer genes controlling valuable agronomic traits. Successful transfer of these chromosomes during hybridization is reliant on meiotic behavior of both wheat and alien chromosomes. When a wheat chromosome, the genes of which are involved in meiotic division regulation, is substituted with an alien one, the presence/ absence of a compensatory effect may be observed in genes on homoeologous chromosomes of relative species and genera. It was shown earlier that wheat chromosome 3B also harbored genes regulating bivalent formation independently of 5В and that Ttzip4-B1 was co-expressed with orthologs of meiotic gene

In our study, we have investigated meiotic MT cytoskeleton dynamics and chromosome behavior in the 3R(3B) line with wheat chromosome 3В substituted with rye chromosome 3R. The effect of 3R(3B) substitution manifested itself not only in reduced chiasmata count compared to the S29 variety (34.9 ± 0.62 and 41.92 ± 0.38, respectively), but also in a series of anomalies in MT dynamics and chromosome behavior in the first and second divisions. The disturbances had to do with MT nucleation at kinetochores, MT convergence at meiotic spindle poles, C-shaped spindle formation, cell wall construction, cytomixis, as well as asynchronous second division and the presence of dyads at telophase II. Thus, the results obtained show that chromosome 3В of the Saratovskaya 29 variety is involved in regulation of a series of meiotic processes, and rye chromosome 3R lacks a genetic compensatory ability to functionally replace 3B in terms of normal meiotic progression

## Conflict of interest

The authors declare no conflict of interest.
